# Two Rare Cases in Practice

**Published:** 1893-08

**Authors:** P. L. Hillsman

**Affiliations:** Albany, Ga.


### TWO RARE CASES IN PRACTICE.

BY P. L. HILLSMAN, M. D., ALBANY, GA.

### OCCLUSION OF THE OS UTERI COMPLICATING LABOR AT TERM.

Case I. Mrs. H., age twenty-nine, robust, well-developed, married nine years. Began menstruating in her fourteenth year, and was quite regular until she conceived. Six months prior to conception had some form of uterine disorder, and was under the care of a physician until a short time before she missed her menses.

Gestation progressed without any unusual symptoms to full term. Labor pains commenced about 1 o'clock a. m., and continued until 7 a. m., when I was called to attend her. On arrival found pains frequent and rather of an expulsive character. Digital exploration revealed uterus well descended into pelvic canal, with the ovoid form of vertex presentation, but no os could be found.

After a more minute examination I detected a point somewhat depressed and more attenuated than the surrounding tissue, and being satisfied that it was the os occluded by adhesive inflammation, I proceeded to operate as follows :

An ordinary uterine sound was forced through the most depressed point ; this was followed by uterine dilator, and parts expanded until the index finger could pass. Full dilatation was now gradually accomplished with the fingers, aided by moderate chloroform narcosis, and labor terminated without further assistance at 5 o'clock p. m.

Child was still born, attributable to tedious and prolonged labor. Patient made an uninterrupted recovery, and has since been perfectly regular in her menses.

Remarks. History of this case goes to show that at the time of conception she had endocervicitis, which resulted in adhesion of the parts—there being no menstrual flow to mechanically expand the os.

ABDOMINAL PREGNANCY.

Case 2. E. J., colored, aged forty-two, married; has given birth to five children; last child four years ago. Consulted me April 1, 1892, with history as follows:

Fifteen months ago missed her menses, symptoms of pregnancy followed. She also thought that she experienced quickening, and made ready for the event of parturition. Having gone several months over her time without any symptoms of labor she consulted a physician, who informed her that she had uterine fibroid, and that she would in all probability get relief after her courses left her. She was comparatively well at the time, and continued her vocation (that of an ordinary hand in the field) until about one month before consulting me, when, owing to feeble health, she was compelled to give up her work.

Subject, at the time of her visit, was emaciated, complained of gastric disturbance, said that she felt as if her stomach was not large enough to hold sufficient food to satisfy her hunger, and vomiting ensued if a moderate meal was taken. Abdomen enlarged—presenting the appearance of pregnancy at term.

On examination I found the uterus anteverted, and with difficulty passed a curved sound three and one-half inches. The tumor gave some evidence of semifluctuation.

Case was diagnosed as ovarian cyst, possibly malignant in character, as patient had lost flesh rapidly, and the gastric disorder led me to suspect similar trouble in stomach. Operation urged, but owing to her husband being actively engaged in crop it was deferred for the time. The patient was placed on tonic course, and nutritious diet ordered in small quantities, and often repeated.

July 1st patient returned and insisted on the opperation; stated that she had gained some strength shortly after first visit; had her menses to come on twice, and was hopeful of recovery, but for the past two weeks had begun to fail again, and vomit her food.

Operation July 4, 1892. Under strict antisepsis abdominal incision was made from the umbilicus down near to symphysis; walls very attenuated and completely adherent to sac. After freeing adhesion on left side about one inch from median line, peculiar pouch was discovered, which was at first very puzzling. On further dissection, however, it was found to be a reflection or folding of the sac on itself. This was caused by a partial absorption of the liquid contents of the sac, and the resilient abdominal muscles caused an overlapping of the flaccid walls. The sac having been freed as far as the incision would allow a trocar was introduced and the liquid contents withdrawn. Only about one pint of thick caseous matter passed, the appearance of which rather strengthened my fear of malignancy.

Finding it impossible to bring the mass through this opening, the incision was enlarged through and above the umbilicus about four inches. Further effort was now made to free the sac from its intimate adhesion to the adjoining viscera, which being found impracticable was abandoned. The sac was now incised and (to my great relief) was found to contain a well preserved child weighing seven pounds. This, with its shriveled placenta, was readily extracted without any hemorrhage. The freed portion of the sac was removed and edges stitched to abdominal walls, drainage tube placed in abdominal cavity at lower point of incision and sac loosely packed with iodoform gauze. Dressing was completed with thick wadding of iodoform cotton covered by gauze and bandage.

Patient rallied well from the operation, and began to cry piteously for food. She was allowed one teaspoonful of Wyeths Meat Juice every four hours, and small quantities of crushed ice.

July 5th, temperature 99½ degrees. Slept well during night from hypodermic morphia. Still clamoring for solid food.

July 6th, temperature 100½ degrees. Allowed milk with lime water in small quantities, and egg albumen water.

July 7th, 10 o'clock a. m., found patient in collapsed state, with great pain in bowels ; abdomen much distended with flatus, more particularly over region of stomach. Breath exhaled an odor of decayed eggs. On questioning the attendant, her husband, he acknowledged that he had given her three eggs for supper on previous night, and other things in proportion. He said he did not think she would ever get up without getting more to eat. Stomach tube was inserted and about half gallon of offensive matter removed and organ cleansed with warm water. Dressing was removed and wound found in good condition ; very little discharge having taken place since operation.

Patient did not rally from the state of collapse, and died at 6 o'clock p. m. Her body was removed same night to the country, and consequently no autopsy was made.

Remarks. The progress of this case up to third day led me to hope for a favorable result, and the unhappy termination should be justly attributed to the pain and distress incident to the heavy meal of eggs, etc., taken for supper.

The condition of the wound would exclude septic poison as a possible cause of death.

